# Compaction Behavior of Isomalt after Roll Compaction

**DOI:** 10.3390/pharmaceutics4040494

**Published:** 2012-09-27

**Authors:** Julian Quodbach, Johanna Mosig, Peter Kleinebudde

**Affiliations:** Institute of Pharmaceutics and Biopharmaceutics, Heinrich-Heine-University, Universitaetsstrasse 1, 40225 Duesseldorf, Germany

**Keywords:** isomalt, roll compaction, work hardening, recompression, compactibility

## Abstract

The suitability of the new isomalt grade galenIQ™ 801 for dry granulation and following tableting is evaluated in this study. Isomalt alone, as well as a blend of equal parts with dibasic calcium phosphate, is roll compacted and tableted. Particle size distribution and flowability of the granules and friability and disintegration time of the tablets are determined. Tensile strength of tablets is related to the specific compaction force during roll compaction and the tableting force. In all cases, the tensile strength increases with raising tableting forces. The specific compaction force has a different influence. For isomalt alone the tensile strength is highest for tablets made from granules prepared at 2 kN/cm and 6 kN/cm and decreases at higher values, *i.e.*, >10 kN/cm. Tensile strength of the blend tablets is almost one third lower compared to the strongest tablets of pure isomalt. Friability of pure isomalt tablets is above the limit. Disintegration time is longest when the tensile strength is at its maximum and decreases with higher porosity and lower tensile strengths. Isomalt proves to be suitable for tableting after roll compaction. Even though the capacity as a binder might not be as high as of other excipients, it is a further alternative for the formulation scientist.

## 1. Introduction

Isomalt is a polyol derived from sucrose. Advantages of this excipient are its sweet taste, which is supposed to allow taste masking, the low glycemic and insulinemic response [1], and the ability to be compacted directly. With regard to infantile patients, also the lack of cariogenicity is important [2]. The marketed products differ mainly in the particle size distribution and the chemical composition. Isomalt consists of varying amounts of 1-*O*-D-glucopyranosyl-D-mannitol dihydrate (GPM) and 6-*O*-D-glucopyranosyl-D-sorbitol (GPS). Since the water solubility of GPS is higher, the marketed products show deviating physicochemical behavior [3]. Ndindayino *et al*. [4,5] investigated the performance of isomalt in direct compression. It was discovered, that isomalt exhibits plastic deformation and elastic recovery mostly in the die. One study determined that isomalt is also an option as diluent for orally disintegrating tablets [6]. Moreover, isomalt was found to be beneficial as excipient for wet granulation with acetaminophen [7]. 

Roll compaction/dry granulation is a frequently used operation in the pharmaceutical industry. As described in literature excipients can lose their ability to form strong tablets after roll compaction/dry granulation [8,9,10]. Due to this phenomenon called work hardening, it is favourable to know how materials behave on recompression. galenIQ™ 801 is a new brand of isomalt consisting of about 75% GPS and 25% GPM. It is supposed to be an easy to granulate, agglomerate and compact filler/binder. This study investigates the preservation of its compactibility after roll compaction.

## 2. Materials and Methods

### 2.1. Materials

Isomalt (galenIQ™ 801, Beneo Palatinit GmbH, Germany), dibasic calcium phosphate anhydrite (DCP, Dicafos C 92-05, Chemische Fabrik Budenheim KG, Budenheim, Germany), fumed silica (Aerosil^®^ 200, Evonik Degussa GmbH, Germany) and magnesium stearate (Bärlocher, Germany) were used. Isomalt and DCP were sieved through a 2000 µm sieve. For the mixture equal parts (*w*/*w*) of isomalt and dibasic calcium phosphate were blended for 10 min in a Turbula mixer (T2C, Willy A. Bachofen AG, Basel, Switzerland).

### 2.2. Methods

#### 2.2.1. Roll Compaction

Two different powders were granulated. The first one was pure isomalt, the second one a blend of equal parts of isomalt and DCP. They were granulated with a Minipactor 250/25 (Gerteis Maschinen + Prozessengineering AG, Jona, Switzerland) equipped with a star granulator and a 1 mm sieve. The star granulator was set to move 120° clockwise and 180° counterclockwise with a gap width of 1 mm and a rotor speed of 40 rpm clockwise and 60 rpm counterclockwise. Speed of the rolls was set to 1 rpm with a gap width of 2.0 mm. Specific compaction forces were 2 kN/cm, 6 kN/cm, 10 kN/cm and 14 kN/cm for isomalt. The isomalt batch produced at 2 kN/cm showed no promising results, thus no DCP blend was roll compacted at this specific compaction force.

#### 2.2.2. Analysis of Powders and Granules

The true density was acquired with a helium pycnometer (AccuPyc 1330, Micromeritics GmbH, Aachen, Germany; *n* = 3). Laser diffraction (Helos, Sympatec GmbH, Clausthal-Zellerfeld, Germany) was used to evaluate the particle size distribution of raw materials, whereas sieve analysis was performed to characterize the granules using a sieve shaker (Vibro, Retsch GmbH, Haan, Germany) for 5 min with an amplitude of 1 mm with 1400 µm, 1000 µm, 710 µm, 500 µm, 355 µm, 250 µm, 180 µm, and 100 µm sieves. The residue was further sieved with an air-jet sieve (AS 200 jet, Retsch GmbH) for 2 min with an under-pressure of 1700 Pa using 90 µm, 63 µm and 32 µm sieves (*n* = 2). The results were interpolated using the software OriginPro 8.5G (OriginLab Corporation, Northampton, MA, USA) to acquire the *x*_10_, *x*_50_ and *x*_90_ values.

The Hausner factor was calculated from tapped and bulk density, measured with a volumetric analyzer (J. Engelsmann AG, Ludwigshafen, Germany; *n* = 3). Also, measurements with a ring shear cell (RST‑01.pc, Dr.-Ing. Dietmar Schulze, Wolfenbüttel, Germany) provided the flow function coefficient (ff_c_) values of granules and powders (*n* = 3) to describe the flow properties.

#### 2.2.3. Tableting and Characterization of Tablets

Prior to tableting, 0.5% magnesium stearate were added to the granules and powders and mixed for 2 min in the Turbula mixer. Ungranulated powders were mixed beforehand with additional 0.5% of Aerosil. All batches were tableted on a rotary die press (Pressima, IMA Kilian, Köln, Germany) set to 15 rpm and compaction forces of 3 kN, 6 kN, 9 kN, 12 kN and 15 kN. Flat faced tablets of a weight of 350 mg and a diameter of 12 mm were produced. Tablet height, weight, diameter and breaking force were determined with a MultiCheck 5.1, friability with a TA friability tester (both Erweka GmbH, Heusenstamm, Germany) according to Ph. Eur. 7.0. Breaking force and tablet size were used to calculate the tensile strength according to Fell and Newton [11]. A DT2 (Sotax, Allschwill/Basel, Switzerland) was used to measure the disintegration time (Ph. Eur. 7.0) in water.

#### 2.2.4. Nomenclature

Batch nomenclature is devised according to the following scheme:

*Granules* *(G) or tablets (T)-isomalt (A) or isomalt-DCP (B)-specific compaction force (kN/cm)_tableting force (kN) (if tableted)*

e.g., T-A-2_15 refers to isomalt tablets roll compacted with 2 kN/cm and tableted with 15 kN.

## 3. Results and discussion

### 3.1. Characterization of Granules

The flowability is characterized in terms of the ff_c _value and the Hausner factor. ff_c _values below 4 describe cohesive behavior, the range from 4 to 10 points to easy-flowing materials, whereas an ff_c _value greater 10 means free-flowability [12]. The results are given in Table 1. Even the lowest specific compaction force of 2 kN/cm improves the flowability of pure isomalt from cohesive (ff_c_ = 3.24 ± 0.02) to easy flowing (ff_c_ = 6.80 ± 0.71). Specific compaction forces of 6 kN/cm and higher produce granules which are all free flowing with ff_c _values above 10. The Hausner factor behaves accordingly, decreasing from 1.33 for the uncompacted isomalt to 1.11 for granules produced with a specific compaction force of 14 kN/cm.

**Table 1:** Flow parameters and particle size percentiles (mean, coefficient of variation <15%, only *x*_10_ of G-A-6, *x*_10_ and *x*_50_ of G-B-6 and *x*_10_ of G-B-14 exceed 15%).

Specific compaction force [kN/cm]	Isomalt granules					Isomalt-DCP granules			
	0	2	6	10	14	0	6	10	14
Hausner factor	1.33	1.21	1.16	1.15	1.11	1.44	1.24	1.15	1.15
ff_c_ value	3.6	6.8	12.1	16.6	16.5	3.2	4.8	10.1	8.4
*x*_10_ [µm]	1.7	25.0	141.5	184.0	186.5	1.0	36.6	37.5	41.0
*x*_50_ [µm]	17.7	546	642	607	617	7.2	556	590	554
*x*_90_ [µm]	44.6	923	934	921	938	31.1	920	927	928

Similar results are obtained for the isomalt-DCP batches. The ff_c_ value increases with higher specific compaction forces up to 10 kN/cm, yet displays a slight drop below the free flowing mark of 10 with an ff_c_ value of 8.41 ± 0.06 at 14 kN/cm. This can be due to brittle behavior of the excipient after being compacted with the highest specific compaction force, thus creating more fines. In this case, the ff_c_ value is a more discriminating and accurate indicator for the flowability than the Hausner factor, which is 1.15 for both, the 10 kN/cm and 14 kN/cm batch.

The increased flowability can be explained by the strong increase in particle size due to roll compaction (Table 1). Laser diffraction reveals an *x*_90_ of 44.64 ± 1.16 µm for pure isomalt and 31.05 ± 1.02 µm for the isomalt-DCP blend. After roll compaction the *x*_90_ of pure isomalt as well as isomalt-DCP granules is in all cases above 900 µm. Yet, the isomalt-DCP granules have only small *x*_10_ values of around 37 µm which explain the lower ff_c_ values compared to pure isomalt granules. Only the 2 kN/cm batch of isomalt granules has an equally small x_10_ value, specific compaction forces ≥6 kN/cm lead to an *x*_10_ of ≥140 µm. The *x*_50_ value increases from 546 µm to 642 µm in the same step. The largest *x*_50_ value is at 6 kN/cm for isomalt granules (642 µm) and at 10 kN/cm for isomalt-DCP granules (590 µm). It decreases again at higher specific compaction forces, indicating a maximum of plastic deformation at these forces. The material begins to behave brittle as soon as the specific compaction force is raised and the particle size decreases. 

### 3.2. Characterization of Tablets

**Figure 1:**
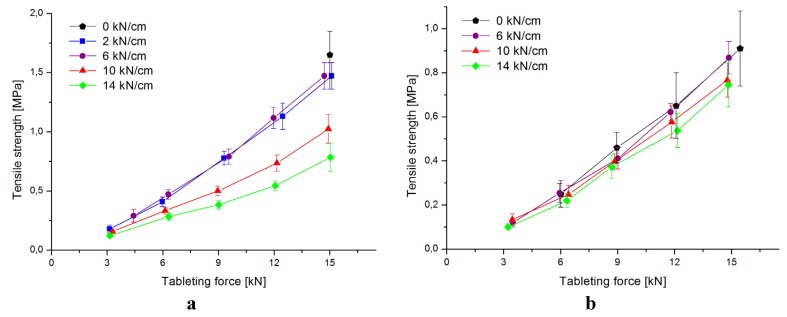
(**a**) Compactibility of isomalt tablets; (**b**) Compactibility of isomalt-DCP tablets. (mean ± SD, *n* = 10).

As a measure of compactibility, the tensile strength was plotted against the tableting force. As depicted in Figure 1a, it is evident that large specific compaction forces (*i.e.*, ≥10 kN/cm) result in weaker tablets of pure isomalt. T-A-2_15 and T-A-6_15 have tensile strengths up to 1.5 MPa, however tablets of granules produced with a specific compaction force of ≥10 kN/cm have lower tensile strengths. Batch T-A-14_15 displays a drop to below 1 MPa. Only one batch of ungranulated isomalt could be produced (T-A-0_15) because lower tableting forces lead to soft and easily breaking tablets. The difference in tensile strength between ungranulated isomalt and isomalt granulated at low specific compaction forces is small, indicating only little work hardening.

The tablets produced with the isomalt-DCP blend behave differently (Figure 1b). The differences in tensile strength are small across all compaction forces. Yet, the overall tensile strength is in all cases lower than the one of pure isomalt tablets. The strongest tablets made from granules are compacted with a specific compaction force of 6 kN/cm and tableted with 15 kN (T-B-6_15). But even in this case, the tensile strength is in average below 0.9 MPa.

These results mirror in the friability test results. None of the isomalt-DCP tablet batches passes the test. It can be assumed that the brittle behavior of DCP is dominant, thus causing weaker tablets. Pure isomalt tablets roll compacted at 14 kN/cm were affected most from work hardening, as these tablets also fail the test (Table 2). The friability test is successful for tableting forces higher than 9 kN and specific compaction forces of 2 kN/cm and 6 kN/cm. Also, batch T-A-6_9 passes the test.

**Table 2:** Friability test results of isomalt tablets according to Ph. Eur. 7.0.

	2.0 kN/cm	6.0 kN/cm	10.0 kN/cm	14.0 kN/cm
3 kN	not passed	not passed	not passed	not passed
6 kN	not passed	not passed	not passed	not passed
9 kN	not passed	0.91%	not passed	not passed
12 kN	0.62%	0.75%	not passed	not passed
15 kN	0.74%	0.60%	not passed	not passed

Ritschel defines the approximate target value of breaking force as 10 N times the diameter in millimeter [13]. The tablets compressed in this study have a diameter of 12 mm and a height of about 2.3 mm in average, implying a target breaking force of roughly 120 N. Even the strongest produced tablets show a breaking force of only 40 N.

**Figure 2:**
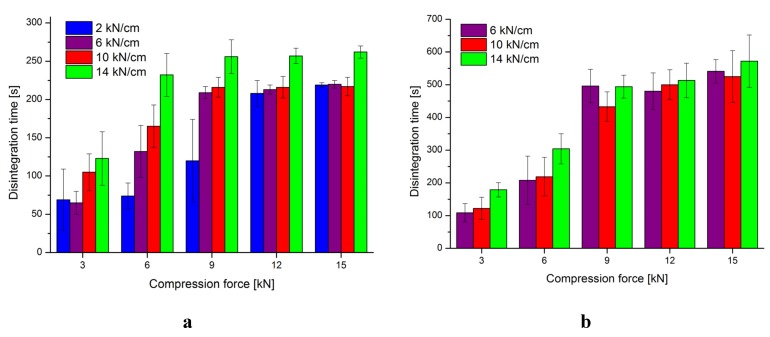
(**a**) Disintegration time of isomalt tablets. (**b**) Disintegration time of isomalt-DCP tablets. (*n* = 6, mean ± SD).

Furthermore, the disintegration time according to Ph.Eur. was measured. In most cases, the tablets disintegrate as expected. The higher the specific compaction and tableting force, the longer the tablets need to disintegrate (Figure 2a,b). Isomalt-DCP tablets tend to disintegrate slower than isomalt tablets in most cases. This can be due to the very low water solubility of DCP.

During the dry granulation and tableting processes, the intraparticular and interparticular porosities are affected. Whereas the intraparticular porosity is mainly altered during roll compaction, the interparticular porosity decreases with increasing tableting forces. This explains why some batches disintegrate slowly, even though the tableting force was kept low. For example, T-A-14_3 disintegrates within the same time range as T-A-2_9, albeit the tableting force is tripled. The intraparticular porosity is too small to allow quick wicking. The tensile strength of T-A-2 batches is similar to that of T-A-6 batches, yet the tablets disintegrate faster at 6 kN and 9 kN. It can be assumed, that the higher intraparticular porosity due to the lower specific compaction force facilitates the entry of water and therefore accelerates disintegration. The data suggest that the specific compaction force has a higher impact on the disintegration time than the tableting force. Yet, the differences in disintegration time decrease at compression forces ≥9 kN. The impact of the specific compaction force also decreases with increasing compressions forces. Only pure isomalt roll compacted with 14 kN/cm displays higher disintegration times compared to the other batches.

## 4. Conclusion

Work hardening also affects the new isomalt brand galenIQ™ 801. Increasing the specific compaction force during dry granulation resulted in decreasing tensile strengths of produced tablets. However, it is still shown that isomalt is suitable for dry granulation and further tableting. A specific compaction force of 6 kN/cm seems to be the best setting for roll compaction, resulting in freely flowing granules as well as tablets with the highest tensile strength.

It must be mentioned, however, that the compressed tablets were not comparable with marketed products in terms of size and composition. The thickness ranged between 1.90 mm and 3.05 mm for a 12 mm diameter, which is unusually flat and resulted in weak compacts not on a par with standard tablets. The tablet size was particularly low merely because a limited amount of substance was available. The friability test failed due to the fact that the tablets broke apart rather than because of abrasion. Whether tablets with a common drug load and thickness are sufficiently strong needs to be further investigated.
